# Spatial Optimization of Residential Care Facility Configuration Based on the Integration of Modified Immune Algorithm and GIS: A Case Study of Jing’an District in Shanghai, China

**DOI:** 10.3390/ijerph17218090

**Published:** 2020-11-03

**Authors:** Min Cheng, Xiao Cui

**Affiliations:** 1 Department of Management Science and Engineering, School of Management, Shanghai University, Shanghai 201900, China; chengmin@shu.edu.cn; 2 Department of Construction Management and Real Estate, School of Economics & Management, Tongji University, Shanghai 200092, China

**Keywords:** residential care facility (RCF), spatial optimization, facility configuration, multi-objective, geographic information systems (GIS), modified immune algorithm (MIA)

## Abstract

As the population is aging rapidly, the irrationality of residential care facility (RCF) configuration has impacted the efficiency and quality of the aged care services so significantly that the optimization of RCF configuration is urgently required. A multi-objective spatial optimization model for the RCF configuration is developed by considering the demands of three stakeholders, including the government, the elderly, and the investor. A modified immune algorithm (MIA) is implemented to find the optimal solutions, and the geographic information system (GIS) is used to extract information on spatial relationships and visually display optimization results. Jing’an District, part of Shanghai, China, is analyzed as a case study to demonstrate the advantages of this integrated approach. The configuration rationality of existing residential care facilities (RCFs) is analyzed, and a detailed recommendation for optimization is proposed. The results indicate that the number of existing RCFs is deficient; the locations of some RCFs are unreasonable, and there is a large gap between the service supply of existing RCFs and the demands of the elderly. To fully meet the care demands of the elderly, 6 new facilities containing 1193 beds are needed to be added. In comparison with the optimization results of other algorithms, MIA is superior in terms of the calculation accuracy and convergence rate. Based on the integration of MIA and GIS, the quantity, locations, and scale of RCFs can be optimized simultaneously, effectively, and comprehensively. The optimization scheme has improved the equity and efficiency of RCF configuration, increased the profits of investors, and reduced the travel costs of the elderly. The proposed method and optimization results have reference value for policy-making and planning of RCFs as well as other public service facilities.

## 1. Introduction

In recent years, China’s population is aging rapidly due to the one-child policy and a reduced mortality rate. Shanghai, one of the largest cities in China, is facing a severe challenge with regard to the aging population. During the past few decades, Shanghai has gone through a dramatic demographic transition from high fertility and high mortality to low fertility and low mortality, inevitably leading to rapid aging of the population. The registered elderly population (aged 60 and over) accounted for 35.2% of the total population of Shanghai in 2019, which is a 56.5% increase over the proportion of the elderly population in 2010 [1]. Residential care facilities (RCFs) play an important role in caring for the elderly. The Special Plan for the Layout of RCFs in Shanghai [2] issued by the Shanghai local government proposed the planning goal that the number of beds in RCFs in each district of the central urban areas should be no lower than 2.5% of the registered elderly population. However, at present, the total beds offered by RCFs in Shanghai are still insufficient, and the spatial layouts of RCFs in some districts are unreasonable [3,4]—directly reducing the efficiency and quality of its services and affecting the improvement of urban elderly care services. Therefore, it is necessary to optimize the configuration of RCFs to provide more efficient and equitable public services.

The existing research on RCFs has mainly focused on two aspects: One aimed at analyzing the demands on RCFs from the perspective of the willingness, preference, economic conditions, and living conditions of the elderly [5,6,7,8]. The other has concentrated on the development status of RCFs, including accessibility, satisfaction, and influence factors [9,10,11,12,13]. However, little attention has been paid to the optimization of RCF configuration. Existing research on the configuration of RCFs has been primarily conducted from three aspects. Firstly, some studied the configuration status by using proportional indicators such as the number of beds per 100 elderly people and geographical area each covered per bed and then put forward some suggestions for the configuration of RCFs from the perspective of urban planning [14,15]. Although the proportional indicators are easy to calculate, they do not reflect some of the other crucial factors affecting the configuration of RCFs, such as the quantity, scale, or layout of RCFs. These factors also greatly impact the effectiveness of the supply of elderly care services. Secondly, some studies were conducted from the perspective of sociology. Questionnaires or interviews were used to analyze the care demands of the elderly on RCFs [16,17]. These kinds of studies focused on the rationality of the quantity of RCFs and the quality of services, but less on the spatial layouts of RCFs. The third group used GIS-based technology to analyze the rationality of RCF locations of RCFs and make suggestions for optimization from the perspective of geography [18,19,20]. This type of study was more concerned with the spatial analysis of RCFs than their quantity. To sum up, the existing research still has some limitations. On the one hand, they only considered the demands of the elderly without involving other stakeholders. On the other hand, they only analyzed locations or the quantity of RCFs separately and independently without combining the two and other aspects. In fact, the configuration of RCFs involves multiple stakeholders, such as the elderly, the government, and the investor. When configuring RCFs, it is necessary to comprehensively consider the demands of various stakeholders and the impact of multiple factors. Therefore, it is essentially a multi-objective nonlinear dynamic decision-making problem with complex constraints. Multi-objective optimization problems are commonly solved by intelligent algorithms such as a genetic algorithm (GA), particle swarm optimization (PSO), and immune algorithm (IA), etc. Although GA can quickly perform a global search, it requires complex coding and has the disadvantages of slow convergence rate, easy to premature convergence, and low accuracy when dealing with complex problems [21]. For PSO, it has deficiencies in premature convergence, weak local searchability, and low accuracy of solutions in the early stage [22]. IA, a new computational method inspired by the biological immune system, was first proposed by Jerne [23]. As a heuristic method, it can learn new information, recall previously learned information in a highly decentralized fashion, and can tackle complex real-world problems. Many studies have demonstrated that IA possesses certain attractive immune properties that allow it to escape from local optima and avoid premature convergence [24,25]. However, the IA still has the disadvantages of a slow convergence rate in the late search stage and low accuracy. Therefore, it is necessary to make modifications to improve the performance of IA [26].

In addition, the configuration of RCFs is a complex issue of decision-making and spatial optimization, in which both the optimization of quantity and layout should be considered simultaneously. Although the intelligent algorithms can be utilized to study the complex systems and dynamic behavior, they still have some shortcomings in spatial optimization. Fortunately, geographic information system (GIS) can provide a variety of spatial data for spatial decision and has the advantage of spatial visualization. As a result, the modified immune algorithm (MIA) and GIS are integrated to analyze and solve the optimization problems in spatial decision-making in this study, allowing their respective advantages to work in complement with each other and help to simultaneously optimize the quantity, locations, and scales of RCFs. Moreover, this approach can give rise to better solutions for multi-objective optimization configuration of RCFs compared with application of IA alone, while there are as yet no published studies on the integration of both techniques as an assistant decision-making tool for public service facility optimization. The optimization problem of RCF configuration belongs to an interdisciplinary field involving computer science, operations research, geography, and planning. We introduce complex spatial decision theory and swarm intelligence optimization theory as the theoretical framework, and planning control theory, location theory, and spatial structure theory as the research foundations. The specific research question, research methods, and procedures are described as follows: To optimize the configuration of RCFs, considering the demands of the three stakeholders of the government, the elderly, and the investor, an optimization model for RCF configuration is constructed. By taking the Jing’an District of Shanghai as an example, the configuration rationality of the existing RCFs is analyzed firstly, and then the optimization model is solved and analyzed by integrating MIA and GIS. According to the results, some optimization suggestions are further proposed to provide references for the planning of RCFs. The approach combining GIS and optimization techniques proposed in this study can also be used for the optimization of the configuration of other public service facilities in practice and can enrich the method of public service facility planning systems in theory. The remaining part of this study is organized as follows. In the following section, the optimization model for RCF configuration is developed, and the MIA is proposed. Followed by that, by taking the Jing’an District of Shanghai in China as an example, the MIA is used to solve the developed optimization model, and GIS is integrated with MIA for the spatial optimization analysis of the RCFs. The last two sections discuss the calculation results and conclude the study, respectively.

## 2. Methods

### 2.1. Optimization Model

According to the rules for the locations of RCFs in the Special Plan for the Layout of RCFs in Shanghai, a set of candidate sites for RCFs were identified through GIS tools (see Section 3.3). Given a set of population centers and a set of candidate sites for RCFs, the optimization of RCF configuration was to identify a reasonable number of RCFs, which have the optimal scales and locations among the candidate sites to fully meet the care demands of the elderly in corresponding population centers. The configuration of the RCFs involved the decision-making behaviors of the government, the elderly, and the investor. Equity and efficiency were the main goals of the government when allocating public service resources. Therefore, governmental departments will try to reasonably configure the RCFs to balance equity and efficiency and meet the principle of maximizing equity and efficiency. In terms of the convenience and cost of travel, the elderly hope that RCFs were adjacent to their own dwellings or their children’s, which was in line with the principle of minimizing travel costs. Investors expect to maximize their profits with minimal investment, and their decisions followed the principle of maximizing profits. To this end, the optimization model was established by maximizing the equity and efficiency of government-configured service facilities, minimizing the travel costs of the elderly, and maximizing the profits of investors as the objectives of the RCF configuration in this study. Specific mathematical descriptions of the model are as follows:

#### 2.1.1. Maximizing Equity

The disparities of accessibility to RCFs can reflect the equity of configuration. The smaller the disparities of accessibility are, the better the equity will be. It can be calculated as follows [27]:

(1) $$min\;\;\;\overset{I}{\underset{i=1}{\sum }}{\left(A_{si}-a\right)}^{2}$$

(2) $$a=\overset{I}{\underset{i=1}{\sum }}\frac{w_{i}}{W}A_{si}=\frac{V}{W}$$

where *i* is a population center (*i* = 1,2,...*I*), *I* is the total number of the population centers, *A_si_* is the accessibility from the *i*th population center to the nearest RCF after optimization, *a* is the weighted average accessibility, *V* is the total number of beds of all RCFs, *w_i_* is the demands of the *i*th population center, namely the number of the elderly who need the service of RCFs, *W* is the total demands of all population centers. The commonly-used improved gravity-based method is used to calculate the accessibility as follows [28]:

(3) $$A_{si}=\overset{J}{\underset{j=1}{\sum }}\frac{v_{j}d_{ij}{}^{-\beta }}{\sum_{i=1}^{I}w_{i}d_{ij}{}^{-\beta }}Y_{j}$$

where *j* is a candidate site of the RCFs (*j* = 1,2,...*J*), *J* is the total number of candidate sites, *v_j_* is the number of beds available for the *j*th candidate site, *d_ij_* is the travel distance between the *i*th population center and the *j*th candidate site, *β* is the distance-decay parameter, *Y*_j_ is the logical variable, *Y_j_* = 1 if a candidate site is chosen as the site with the best location, its location meets the planning requirements of The Special Plan for the Layout of RCFs in Shanghai and makes the optimization results of RCF configuration be the best. Otherwise, *Y_j_* = 0.

#### 2.1.2. Maximizing Efficiency of Configuration

The efficiency of configuration can be measured by the mean value of accessibility change rates after optimization. The more the mean value of the change rate is, the higher the configuration efficiency will be. It can be estimated as follows:

(4) $$max\;\;\;\frac{1}{I}\overset{I}{\underset{i=1}{\sum }}\frac{A_{si}-A_{oi}}{A_{oi}}$$

where *A_oi_* is the accessibility from the *i*th population center to the nearest RCF before optimization. 

#### 2.1.3. Minimizing Travel Costs

To estimate the travel costs, we used the sum of the product of the demands of each population center and the travel distance from the population center to its nearest RCF [29]. It is calculated as follows:

(5) $$min\;\;\;\overset{J}{\underset{j=1}{\sum }}\overset{I}{\underset{i=1}{\sum }}w_{i}d_{ij}Z_{ij}$$

where *Z_i_*_j_ is the logical variable, which indicates the service allocation relationship between *i*th population centers and RCFs. If the care demands of the *j*th population center are met by the RCF candidate site, then *Z_ij_ = 1*; Otherwise, *Z_ij_ = 0*.

#### 2.1.4. Maximizing Profits

In general, profit is the difference value between income and cost. The mean value of the profits of all RCFs is used to measure investors’ profits. It is written as:

(6) $$max\;\;\;\frac{1}{J}\overset{J}{\underset{j=1}{\sum }}R_{j}Y_{j}$$

(7) $$R_{j}=\left(c_{0}-c_{1}\right)\overset{I}{\underset{i=1}{\sum }}w_{i}Z_{ij}$$

where *Rj* is the profits of the *j*th RCF candidate site, *c_0_* is the average bed charge, *c*_1_ is the average cost of running a bed. According to the survey results and the actual consumption situation of Jing’an District in Shanghai, *c*_0_ ≈ *2050* RMB/month (around $292 USD/month), *c*_1_ ≈ *1250* RMB/month (around $178 USD/month). 

In addition, some constraints should be considered in this optimization problem:

(1) The number of RCFs selected from candidate sites should be equal to a predefined number *p*, namely:
  (8) $$\overset{J}{\underset{j=1}{\sum }}Y_{j}=p$$
(2) The elderly in every population center can get the service from a RCF, namely:
  (9) $$\overset{J}{\underset{j=1}{\sum }}Z_{ij}=1\;\;\;i=1,2,3\cdots I$$
(3) For each selected RCF candidate site, it will serve at least one population center, namely:
  (10) $$Z_{ij}\leq Y_{j}\;\;\;i=1,2,3\cdots I\;\;\;j=1,2,3\cdots J$$
(4) According to the Special Plan for the Layout of RCFs in Shanghai, RCFs are divided into 3 types: small-sized (the number of beds should be less than 100), medium-sized (the number of beds should be between 100 and 300), and large-sized (the number of beds should be between 300 and 500), and the RCFs of Jing’an District, which is located in the downtown area of Shanghai, should be small-sized or medium-sized, and the number of beds here should be between 0 and 300, namely:

(11) $$0<v_{j}Y_{j}\leq 300\;\;\;j=1,2,3\cdots J$$

### 2.2. Modified Immune Algorithm

Immune Algorithm (IA) is a new kind of intelligent search algorithm inspired by the biological immune system. By generating new antibodies and the communication between antibodies in the immune network, the immune system has advantages in learning, memory, and discrimination between self and non-self. IA can be used to solve various engineering problems by simulating the behavior of immunological processes of the immune system [30], namely, the encoded solutions of the problem are treated as artificial antibodies, and the immunological processes powered by clonal selection and maturation will be implemented on them. In the IA, we randomly generated a certain number of antibodies that met the constraints at first and selected some excellent antibodies from them with a certain probability for cloning, and then crossed and mutated these cloned antibodies. As a result, the excellent antibodies of the first generation and these cloned antibodies became the next generation of antibody groups as memory cells. The above iteration was repeated until a global optimal solution was obtained. In the clonal selection process, the cloning proportion of each antibody was based on its affinities, including the affinity between the antibody and antigens and the affinity between itself and other antibodies. The former allowed the well-performed antibodies to be retained, while the latter ensured the diversity of the antibody population. In the iterative processes of IA, the mutation operator was introduced to make the antibodies evolve and adapt to the optimization problem [31].

In the IA, the antigens that invade the immune system represent the multi-objective functions, and the antibodies produced by the immune system represent the feasible solutions of the multi-objective functions. The affinity between each antigen and antibodies represents the degree of approximation between the feasible and the optimal solution, which reflects the ability of an antibody to recognize the antigens. The greater the affinity is, the stronger the antibody’s ability to recognize the antigens will be. To obtain the total affinity value between each antibody and all antigens, an adaptive weighting method is employed to construct a comprehensive objective function for solving the multi-objective problem. The reciprocal of the comprehensive objective function is the total affinity, which is written as [32]:

(12) $$A\left(k\right)=\frac{1}{F\left(x\right)}=\frac{1}{f\left(x\right)r\left(x\right)}$$

where *A*(*k*) is the total affinity value of the antibody *k*, *F*(*x*) is the comprehensive objective function, *f*(*x*) is the standardized weighted form of all the objective functions, *r*(*x*) is the adaptive penalty function. *f*(*x*) and *r*(*x*) is written as follows:

(13) $$f\left(x\right)=\overset{4}{\underset{l=1}{\sum }}\lambda_{lk}f_{lk}^{\prime }\left(x\right)$$

(14) $$r\left(x\right)=1-\frac{1}{4}\left[\frac{\Delta b_{kg}\left(x\right)}{\Delta b_{kg,max}}{\left[\right]}^{\eta }\right]$$

where *l* is the objective function, *λ_lk_* is the *k*th antibody’s weight of *l*th objective function, *f^′^_lk_*(*x*) is the *k*th antibody’s standardized form of *l*th objective function, *g* denotes the constraints, *Δb_kg_*(*x*) is the *g*th constraint violation of the *k*th antibody, *Δb_kg, max_*(*x*) is the *g*th maximum constraint violation of the *k*th antibody, *η* is the penalty exponent.

Although with using the IA, premature convergence can be avoided, and the global optimal solutions can be obtained easily, it still has some drawbacks, such as slow convergence rate and low search accuracy. Therefore, it is necessary to improve the standard IA further to obtain better optimization results. Since the selection operator and mutation operator can affect the searchability and calculation accuracy considerably [33,34], and diversity is also the key to obtain the global optimal solutions [35], we modified the standard IA as follows.

#### 2.2.1. Variable Threshold for Selection Operator 

In the standard IA, the threshold was used to determine whether the antibodies can be selected for cloning, and its value was usually fixed, which reduced the convergence rate of the algorithm to some extent. Therefore, a variable threshold was introduced to accelerate the convergence rate. A relatively small threshold was set in the early search stages, thus that the probability of antibodies with great affinity and low antibody concentrations being selected and cloned could be greatly increased. In the late search stage, a relatively large threshold was set to greatly reduce the selection and clone opportunities for antibodies with great affinity and low antibody concentrations. In this way, the computational efficiency of the algorithm was improved. The variable threshold is as follows [33]:

(15) $$\sigma =\{\begin{array}{cc} a & t\leq t_{ave}\\ a & sin(\frac{\pi }{2}\times \frac{t_{max}}{tave_{max})\;\;\;t>t_{ave}} \end{array}$$

where *a* is the adjustment coefficient of the threshold, in general, *a* = 0.3, *t* is the current iteration times, *t_ave_* is the average iteration times, *t_max_* is the maximum of iteration times. 

#### 2.2.2. Guo Elite Mutation Operator

As a monoclonal antibody of mutation, the mutation operator was applied to change affinity values and implement local search. In the standard IA, the method of exchanging mutation was generally used, however, it had the disadvantages of the small search range and low efficiency. It was necessary to increase the selection pressure to make the search area more promising, and adopt a more efficient operator to produce an offspring without a burden of many additional calculations and to allow the algorithm to escape from local optima. The Guo elite mutation operator was used in this study to select the antibody with a great affinity for the subspace recombination of multiple elite parents, which greatly ensured the ergodicity of random search. In addition, due to realizing the hybridization of multiple elite parents in this method, the excellent antibodies can be selected accurately and the opportunities for them to breed and survive can also be improved. As a result, the convergence rate of the algorithm is significantly improved. The Guo elite mutation operator is defined as follows [34]:

(16) $$m=\overset{M}{\underset{i=1}{\sum }}\phi_{i}k_{i}$$

where *m* is the new antibodies after mutation, *M* is space of elite parent operator (in general, *M =* 10%*m*), *φ* is an adjustment coefficient, $\sum_{i=1}^{W}\phi_{i}=1$ and −0.5 ≤ *φ_i_* ≤ 1.5, *k_i_* is the *i*th antibody.

#### 2.2.3. Periodically Varying Mutation Probability

Since the mutation probability (MP) can determine the size of the antibody population entering the mutation stage, it has a great influence on the algorithm performance. If MP is low, it is not good for the creation of new excellent antibodies, whereas a high MP does not benefit the retention of excellent antibodies. Inspired by cyclical changes of biological evolution, we varied MP periodically to improve the IA. A large MP was set in the early search stage to generate more excellent antibodies. In the process of evolution, the MP decreased gradually to preserve the excellent antibodies to the greatest extent. In the late search stage, a large MP for some antibodies was set again to improve the searchability of the algorithm. Periodically varying mutation probability was as follows [34]:

(17) $$P_{m}\left(t\right)=\varepsilon \left|\cos\frac{2\pi }{T}t\right|+\gamma$$

where *ε* is the adjustment coefficient of MP, it is usually 0.1, *t* is the current iteration times, *T* is the variation interval, *γ* is the adjustment variable (0< *γ* ≤ *ε*), which can ensure that MP is not 0 when *t* = *KT*/2.

#### 2.2.4. The Perturbations of Global Optima

In the iterative process of the IA, the antibodies are likely to get stuck in local optima and tend to be identical, thus that the diversity of populations cannot be guaranteed. As a result, the algorithm converges prematurely. To solve this problem, the global optima should be perturbed at regular intervals. The perturbation is expressed as [35]:

(18) $$Fit_{best}=Fit_{best}+e^{-\theta \cdot rand^{2}}$$

where *Fit_best_* is the current global optimal affinity, *θ* is the perturbation interval. During the iterative process, the strength of perturbation becomes weaker and weaker, which can ensure that the antibodies are close to the global optima step by step, and the global search ability and the convergence rate of the algorithm are greatly improved.

The main steps of the proposed MIA are as follows:

Step 1: Initialization. The simple decimal encoding is used to initialize an antibody population as a set of randomly generated decision vectors within the predefined feasible solutions. Each antibody represents a candidate solution, which is the sequence of the RCFs selected from the candidate sites.
Step 2: Calculation. In this step, the affinity and the concentration of antibodies are calculated, and some excellent antibodies to store in the memory bank are selected.
Step 3: Memory cell updating. Replace the memory cells whose affinities with antigens are worse with the excellent antibodies. At the same time, for those that have worse affinities with memory cells, their ability is reduced to survive to ensure that the algorithm will not get stuck in local optima.
Step 4: Clonal selection. According to variable threshold for selection operator, a certain number of antibodies with great affinities and low concentrations are selected to be cloned.
Step 5: Mutation. Mutation means random change the permutation of encoding. At this stage, the Guo elite mutation operator is applied to improve the search efficiency, and the periodic varying mutation probability is used to increase the diversity of antibody populations.
Step 6: Perturbation. The global optima is perturbed at set intervals to avoid getting stuck in the local optima.
Step 7: Termination. If $t<t_{\max}$, go to step 2; otherwise, terminate the procedure.

### 2.3. Integration of MIA and GIS

**Figure 1:**
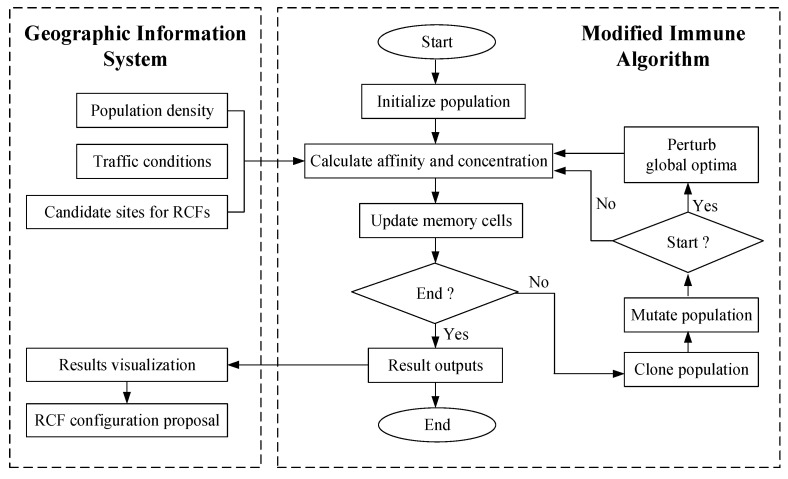
The framework of integrating modified immune algorithm (MIA) and geographic information system (GIS).

The MIA and GIS are integrated and applied to the spatial optimization of RCF configuration, and its implementation framework is shown in Figure 1. Firstly, some GIS tools are adopted to convert geographic information into a variety of spatial data such as population density, traffic conditions, candidate sites for RCFs, and some others needed in this research. By overlapping the population data and layers, and the spatial calculation tool of population density in GIS is used to obtain the population density. Traffic conditions are obtained by the weighted network analysis implemented in GIS (See Section 3.2). Buffer analysis tool and overlap analysis tool in GIS are carried out to generate some candidate areas of RCFs, and then, candidate sites of RCFs are generated from the candidate areas (See Section 3.3). Furthermore, through topological relationship processing, the correctness of the above spatial data is checked. All of the spatial data obtained by the above methods provide a database for the MIA to solve the optimization model. Secondly, by using component GIS, the embedded development method based on ArcObjects (Environmental Systems Research Institute, Redlands, CA, US) is adopted, and ActiveX DLL applications (Microsoft, Redmond, WA, US)based on MIA are developed, thus as to realize the seamless integration of GIS and MIA, thereby achieving efficient data sharing between the two. Through the integration of MIA and GIS, based on the spatial data provided by GIS, the constructed optimization model is solved to obtain the optimal solution. Lastly, GIS is used again to realize spatial visualization of the optimized results obtained by MIA, and map them into a detailed RCF configuration proposal, and multi-criteria decision-making. The integration of MIA and GIS can provide an effective tool for solving complex spatial optimization problems involving multiple-objectives and constraints.

## 3. Model Implementation and Results

### 3.1. Study Area

**Figure 2:**
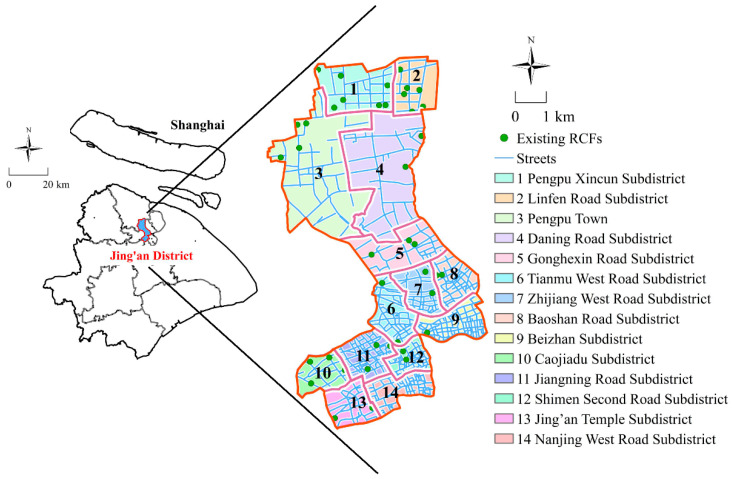
Location of Jing’an District and the spatial distribution of existing residential care facilities (RCFs).

As one of the four municipalities in China, Shanghai is the economic, financial, trade, and shipping center of China. It is situated in the middle of China’s mainland coastline and divided into 16 county-level districts, of which Jing’an District is one of the central districts of Shanghai. With a permanent population of 1.07 million and an area of 37 km^2^, Jing’an District has jurisdiction over 14 sub-districts and 276 residential committees [36]. Due to the large number of the elderly, Shanghai has become the first city in China to become an aging society, and Jing’an District ranks in the top three among Shanghai’s districts for proportion and growth rate of the elderly. According to the demographic data released by the Shanghai Municipal Civil Affairs Bureau, the Shanghai Municipal Statistics Bureau, and the Shanghai Municipal Committee on Aging, there are nearly 326,000 residents aged 60 and over in Jing’an District, accounting for 34.4% of the total population of the district [1]. However, there are only 41 RCFs with 5729 beds in Jing’an District, and the number of beds available is far from meeting the demands of the elderly [37]. The location of the Jing’an District and the spatial distribution of existing RCFs are depicted in Figure 2. Noticeably, there is an obvious disparity in the configuration of RCFs among the various sub-districts in the Jing’an District.

### 3.2. Data Sources

The elderly population data and the location of each residential committee were obtained from the official statistics of Jing’an District [38]. The locations of population centers were defined according to the locations of these residential committees. The data on the number of beds in each RCF were promulgated by the Shanghai Civil Administration Bureau. The road network data were created by the software ArcGIS, and the shortest travel distance from each population center to each RCF was calculated by using the network analysis tool in ArcGIS. Since the road network was dense and the traffic congestion was serious in the Jing’an District of Shanghai, dwell time at the road intersections made up a large part of the residents’ whole travel time [39]. Thus, the road distance alone was not enough to describe the actual travel costs of residents. Therefore, it was necessary to convert the dwell time to the distance according to the corresponding road speed to improve the calculation accuracy of travel costs. By multiplying the corresponding travel speed and dwell time at the intersections, we can get the conversion distances. The actual travel distance was the sum of the shortest distance and the conversion distance. Due to the frequent traffic jam in the central city of Shanghai, the average road speed in rush hours was used as the travel speed.

### 3.3. Definition of Candidate Sites for RCFs

The Special Plan for the Layout of RCFs in Shanghai has made clear rules for the location of RCFs in terms of land use types, location selection, traffic conditions, surrounding environment, and supporting facilities. In order to identify the candidate sites for RCFs, the ArcGIS software is used to carry out buffer analysis and overlap analysis on the spatial vector data such as urban living areas, roads, medical and health facilities, parks, and green spaces to generate some candidate areas of RCFs firstly, and then 810 candidate sites of RCFs are generated from the candidate areas by considering some crucial factors such as the location of residential areas, the layout of existing roads, and the feasibility of housing construction. As a result, in the following research, the best location of RCFs from these candidate sites are all meeting the planning requirements.

### 3.4. Sensitivity Analysis of β

The value of Distance-decay Parameter β has a great impact on calculation results. As summarized in [40], β in existing studies ranges from 0.9 to 2.29, thus nine scenarios that β range from 0.8 to 2.4 are designed for the sensitivity analysis, and the accessibility to RCFs under each β value is computed. By comparing the actual travel time obtained by interviewing residents with the calculated results under different values of β, it is found that when β = 1.8, the calculated results are consistent with the actuality. Therefore, we will discuss the accessibility to the existing RCFs by setting β as 1.8.

However, the locations of RCFs will change greatly after optimization, which will cause the change of the road network between the RCFs and population centers, and the value of β needs to be re-determined. When the number of RCFs is 41, the maximum, minimum, and standard deviations of accessibility under each β value mentioned above are calculated, and the results are as shown in Table 1. It can be seen that as the values of β becoming larger, the distribution of the results tends to diverge, which indicates that the accessibility is sensitive to the value of β and an appropriate β value should be set for better calculation accuracy. When β = 1, the standard deviation is the smallest, namely the disparity of accessibility to the optimized RCFs is the smallest and the equity can be best reflected. Therefore, when the number of RCFs is 41, it is reasonable to set β to 1 for optimization and analysis.

We further calculate the optimal β value when the number of RCFs ranges from 1 to 41. The calculated optimal β values ranges from 0.95 to 1.05, that is, the optimal values of β for different numbers of RCFs are all around 1.0. Therefore, we will set β to 1 for optimization and analysis.

### 3.5. Rationality Analysis of Existing RCFs

The configuration rationality of existing RCFs is analyzed from three aspects, including quantity, scale, and location, as follows.

#### 3.5.1. Quantity

**Figure 3:**
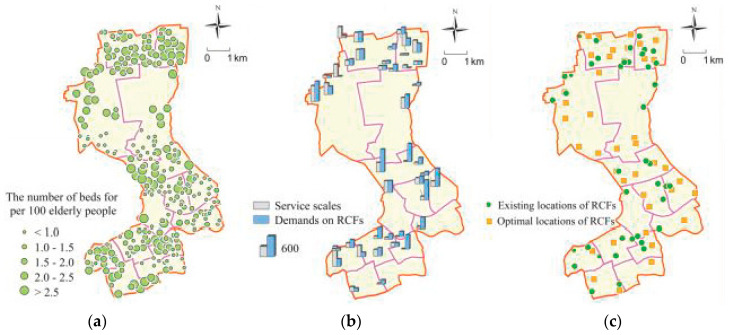
The rationality analysis of the existing RCFs. (**a**) The number of beds per 100 elderly people in each population center; (**b**) the gaps between the service scales and the demands on RCFs; (**c**) the comparison of the existing with optimal locations of RCFs.

According to the Special Plan for the Layout of RCFs in Shanghai, RCFs should provide care services for 2.5% of the elderly population, namely, 8163 beds should be set. However, there are only 41 RCFs with 5729 beds in Jing’an District, and the care demands of the elderly still cannot be met fully. In [41], the effective service radius of an RCF is defined as the travel distance of 1 h, according to Equation (3) (the calculation method of accessibility of RCFs), the number of beds that are available for each population center within the effective service radius can be obtained. As shown in Figure 3a, the locations of circles represent the locations of population centers, and the size of each circle represents the number of beds of RCFs available per 100 elderly people at each population center. From this figure, we can see 72% of population centers have not achieved the goal of 2.5 beds per 100 elderly people proposed in the special plan, which are mainly located in the central and southern part of Jing’an District. According to the findings of [2], in the suburbs of Shanghai, population aging is also severe. From the perspective of the total number of beds in RCFs, the suburbs are facing an even greater gap. However, from the perspective of the number of RCF beds available for per 100 elderly people, the problem of the insufficient configuration of RCF beds is not as severe in the suburbs as that of the central areas, due to the larger land area and the relatively low population density.

#### 3.5.2. Scale

Since it is supposed that each population center is served by its nearest RCF, the number of beds should be set in each RCF to the total number of the elderly who have care demands in the population centers served by corresponding RCFs. The gap between the demands on RCFs and the actual service scales are shown in Figure 3b. It can be seen that some RCFs are oversupplied in scale, which may result in the waste of public resources, while some RCFs are too small to meet the demands of the elderly and require further optimization. This is consistent with the conclusion of [20], which also illustrates the imbalance between the supply and demand of RCFs in Beijing, another metropolis with a serious population aging problem, akin to that of Shanghai.

#### 3.5.3. Location

As mentioned above, there are 41 RCFs in the Jing’an District. By using MIA to solve the configuration optimization model for RCFs, we selected 41 optimal facility sites from the 810 candidate sites. Their locations are shown in Figure 3c. It is clear that many existing RCFs are not in the best locations, which increases the travel costs of residents, weakens the equity of public service, and decreases the profits of investors. It is urgent to optimize the layout of existing RCFs.

Presently, aging in China is very serious, while the task of providing for the elderly is a major challenge. Therefore, since the 18th CPC National Congress, the state has issued a series of policies to promote the development of RCFs. For instance, in 2013, the State Council issued the “Several Opinions on Accelerating the Development of the RCFs” [42], which further reaffirmed the development goals of the eldercare service industry, and clearly stated that it is necessary to vigorously strengthen the construction of RCFs; in 2019, the “Opinions on Promoting the Development of Elderly Care Services” [43] was proposed to accelerate the reform of RCFs and improve their service quality. In addition, as one of the cities facing the most significant aging, Shanghai has formulated a series of special plans to alleviate the contradictions faced by the elderly under the guidance of national policies. Despite the policy support and the government’s attention, there are various problems with the current configuration of RCFs shown above: The number of existing RCFs is deficient, the locations of some RCFs are unreasonable, and there is an imbalance between the supply and demand of RCFs. More scientific methods and theories are needed to propose specific and effective optimization plans for the configuration of RCFs in order to bolster policy implementation and promotion.

## 4. Optimization and Results

The optimization process is as follows:

Step 1: Identify Existing RCFs with Unreasonable Locations and Adjust the Scale of Other RCFs

**Figure 4:**
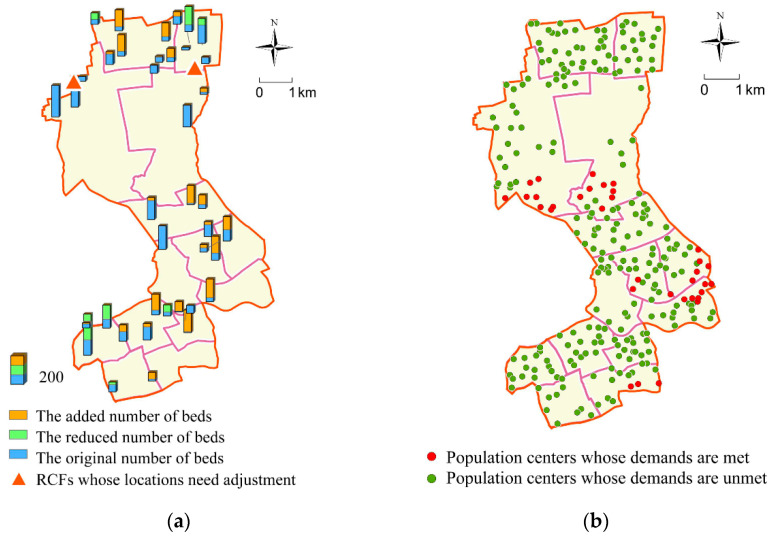
Results obtained by preliminary optimization in step 1. (**a**) The RCFs needed to be optimized; (**b**) the locations of population centers whose demands are met and unmet after preliminary optimization.

According to the above analysis, most of the existing RCFs deviate from the best locations, however, it is unrealistic to adjust the locations of all RCFs. Hence, we only appropriately adjusted a few RCFs with seriously unreasonable locations along the number of beds in some other RCFs. The specific approaches are as follows. By using MIA and GIS to solve and analyze the constructed optimization model, it can be found that there are 2 among the existing 41 RCFs with unreasonable locations requiring adjustment, whose locations are shown in Figure 4a. Next, the numbers of beds in the remaining 39 RCFs are adjusted within the maximum number of beds proposed in the Special Plan for the Layout of RCFs in Shanghai. The difference values of the number of beds between the optimized RCFs and existing RCFs are shown in Figure 4a. After the above optimization and adjustment, the RCFs can only meet the care demands of 6970 elderly people, leaving 1193 elderly people whose demands cannot be met. Therefore, it is necessary to build up some RCFs to meet the demands. The locations of the population centers whose care demands are met and unmet after the preliminary optimization of this step are shown in Figure 4b.

From Figure 4, we can draw the following conclusions. Firstly, RCFs in which the number of beds need to be reduced or whose locations need to be adjusted are mainly located in the northern and southwest marginal areas of Jing’an District, namely Linfen Road Subdistrict, Caojiadu Subdistrict, and the northern of Pengpu Xincun Subdistrict and Pengpu Town. Although population centers are relatively dense in these areas, they are almost small-sized communities, thus the density of the elderly is not as big as other subdistricts. At the same time, the RCFs here are so densely distributed that supply exceeds demand. With regard to the two RCFs requiring adjustment mentioned above, because other RCFs near them are closer to the population center and able to fully meet the demands of the elderly in the vicinity, there are many redundant beds in these two RCFs, causing waste of public resources. Secondly, the number of beds for most RCFs in the central areas of Jing’an District needs to be increased. For some subdistricts in the central of Jing’an District, such as Zhijiang West Road Subdistrict, Baoshan Road Subdistrict, and Beizhan Subdistrict, population centers are densely distributed, and the number of elderly people is large and increasing. Although there are many RCFs here, due to high land prices and the urban planning restrictions, the scales of these RCFs are not large enough to meet the increasing demands of the elderly. Thirdly, after adjusting the number of beds in some RCFs in the south of Pengpu Town, south of Daning Road, and west of Nanjing West Road, the demands of nearby population centers still have not been met. This is mainly because there are few RCFs there, and it is still difficult to meet the huge care demands of elderly people nearby by simply adding beds to existing RCFs. Therefore, it is necessary to establish some new RCFs here to bridge the gap between supply and demand.

Step 2: Determine the Quantity and Locations of RCFs that Need to be Newly Added

**Figure 5:**
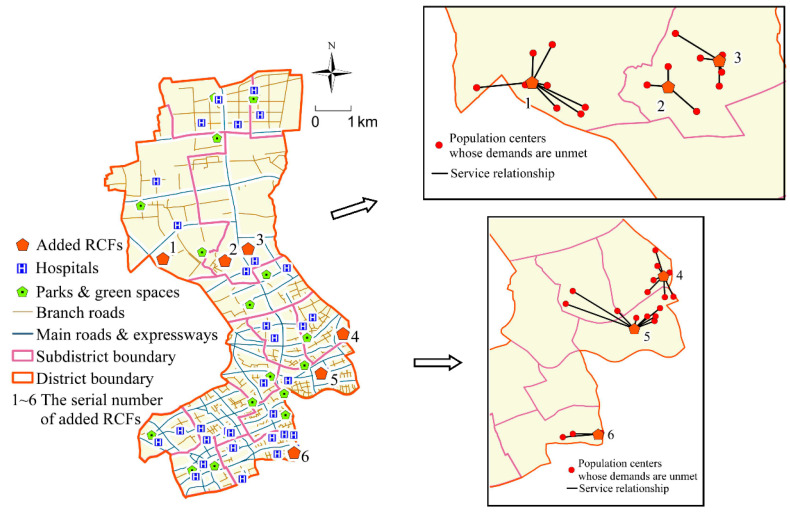
The locations of newly-added RCFs and their service relationship with population centers.

We select some new RCFs from the 810 candidate sites to serve the population centers whose care demands have not been met after the preliminary optimization of Step 1. According to the Special Plan for the Layout of RCFs in Shanghai, the number of beds in each newly-added RCF should not exceed 300. Since there are 1193 elderly people in Jing’an District whose care demands have not been met, at least 4 new RCFs are required. Next MIA and GIS are used to obtain the optimal objective function values for different numbers of RCFs (see Table 2). In this study, the lower the objective function value is, the better the optimization results are. As shown in Table 2, when the number of RCFs is 6, the objective function value is the lowest. It indicates that 6 RCFs should be added to Jing’an District. The locations of the newly-added RCFs are shown in Figure 5.

It can be seen from Figure 5 that the newly-added RCFs are roughly distributed in a densely populated area, which greatly reduces the travel costs of the elderly and improves the equity of residents’ access to public resources. In addition, most of the new RCFs are far away from main roads and highways, close to branch roads, and the transportation is convenient. At the same time, they are located far from noise and pollution sources and close to public facilities such as hospitals, parks, and green spaces, making them suitable for living. This demonstrates that the locations of the new-added RCFs are in line with the planning requirements.

Step 3: Determine the Number of Beds for Each Newly-Added RCF

Since it is assumed that each population center is served by its nearest RCF, the number of beds required for each newly-added RCF is the total number of elderly people in the corresponding population centers who have service demands for RCFs. The service relationship between the newly-added RCFs and population centers is shown in Figure 5. The numbers of beds in newly-added RCFs can be found in Table 3.

## 5. Discussion

### 5.1. Comparison of Optimization Scheme with the Current Situation of RCF Configuration

**Figure 6:**
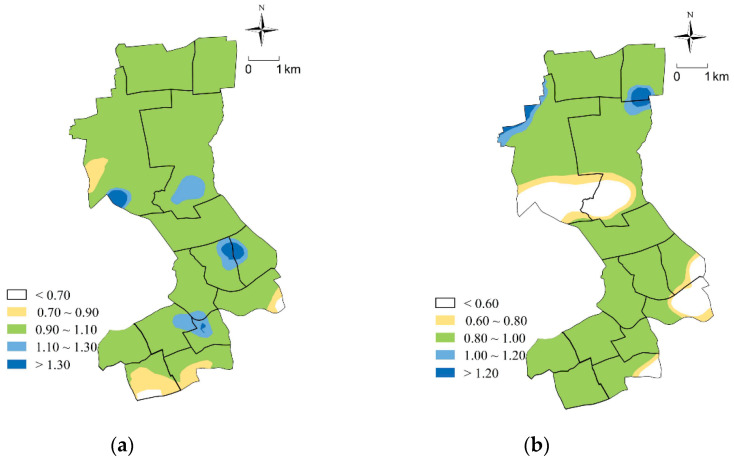
Analysis of optimization results. If there are multiple panels, they should be listed as: (**a**) Ratio of accessibility and the average value; (**b**) ratio of per capita travel costs before and after optimization.

To further illustrate the rationality of the optimization scheme, we compared the optimization scheme with the current situation of RCF configuration. Based on the data of both the optimization scheme and the current scheme, the objective value of each stakeholder was calculated, which is shown in Table 4 and Figure 6. The conclusions we can draw are as follows:

(1) For the government: Firstly, the equity of RCF configuration improves. Table 4 shows that the configuration scheme for RCFs after optimization has 64.23% improvement in equity compared with the current situation. The Gini coefficient of potential service resources available to residents has also dropped from 0.5563 to 0.1225. The Gini coefficient was originally used to measure people’s income equity. In recent years, it has been used by some researchers to reflect the equity of public resource allocation [44]. The lower the Gini coefficient is, the better the equity of resource allocation will be. In addition, the ratio of the accessibility from each population center to the RCF and the average value of the accessibility can also reflect the equity of RCF configuration. The closer the value is to 1, the better the equity of RCF configuration. Figure 6a indicates that 72.83% of the population centers have this ratio indicator between 0.9 and 1.1, which indicates that most population centers can obtain services of RCFs evenly after optimization. Secondly, the efficiency of the RCF configuration improves. The 45 RCFs, including 39 existing RCFs and 6 new RCFs, are able to meet the care demands of all the elderly in Jing’an District after optimization. Table 4 shows that the configuration efficiency of RCFs has been improved by 84.24%. As mentioned above, due to the irrational locations and scales, existing RCFs cannot meet the actual demands, resulting in low service capabilities. When the demand-oriented optimization method is used, the configuration of the RCFs is more balanced, which is more conducive to improving configuration efficiency.
(2) For the investors: Table 4 shows that the economic efficiency of investment has been improved by 29.82%. Therefore, with the optimization of the RCF configuration, the care demands of more and more elderly people are met, while the economic efficiency of investors can also be greatly improved.
(3) For the elderly: Residents’ travel becomes more convenient and efficient. Figure 6b shows that the per capita travel cost is significantly reduced after optimization, and travel efficiency in 99% of population centers has been improved to some degree. From the perspective of the spatial layout of RCFs, there is at least one RCF near each population center. The maximum travel distance from population centers to their nearest RCF is reduced from 2150 m to 1374 m, with the average travel distance reduced from 526 m to 373 m. In addition, the average number of beds available for each population center within a one-hour service radius is increased from 1.5624 to 3.3986, indicating that the optimization scheme has achieved the goal of 2.5 beds per 100 elderly people proposed in the Special Plan for the Layout of RCFs in Shanghai. The above results reveal that the convenience of RCF services after optimization has been improved.

### 5.2. Comparison of the Performance of MIA with Other Algorithms

To further validate the MIA’s rationality and superiority, the performance of MIA was compared with those of the genetic algorithm (GA), particle swarm optimization (PSO), and immune algorithm (IA) on the basis of the same objective functions, data set, and parameter set. Based on the data of the optimization scheme obtained by each algorithm, the objective value of each stakeholder was calculated and firstly standardized according to the adaptive weighting method, and then converted into a comprehensive value of the comprehensive objective in the range of 0 to 1, as shown in Table 5. It is obvious that MIA performs better than other algorithms in terms of equity, efficiency, travel cost, and profits. Meanwhile, it is observed that the objective function values obtained based on the GA, PSO, IA, MIA are 0.7323, 0.8064, 0.6401, 0.6012, respectively. That is, the objective function value of MIA is smaller than that of GA, PSO, and IA by 17.90%, 25.45%, 6.08%, respectively, which also means that the calculation accuracy obtained by MIA is superior to those of GA, PSO, and IA.

The convergence capability of the four methods is further compared. The number of iterations of GA, PSO, IA, and MIA are respectively 650, 680, 700, and 480, which means that the convergence efficiency of MIA is 26.15%, 29.41%, and 31.43% higher than GA, PSO, and IA, respectively. It proves that with regard to the optimization problem of this study, the convergence rate of MIA is faster than that of other algorithms. This result echoes that of the previous study [33], which also shows that the modification of the IA can serve to improve the convergence efficiency.

Additionally, the MIA was also adopted to other districts in Shanghai in our previous study [45], with the optimization results obtained by MIA also better than those obtained by other algorithms, further illustrating the effectiveness and robustness of the MIA.

To sum up, the MIA performs better in terms of calculation accuracy, convergence rate, and robustness.

## 6. Conclusions

The rational configuration of RCFs helps to improve the ability of urban old-age service. Considering the impact of three stakeholders including the government, the elderly, and the investor on the configuration of RCFs, a multi-objective spatial optimization model with complex constraints for RCF configuration is developed based on the goals of maximizing configuration equity and efficiency of government-configured service facilities, minimizing travel costs of the elderly and maximizing profits of investors. An MIA method that improves the performance of standard IA from three aspects, including the selection operator, the mutation operator, and the global optima, is proposed to solve the model. Taking Jing’an District in Shanghai as an example, the rationality of existing RCF configuration in terms of the quantity, scale, and spatial location is analyzed firstly. Then, an optimization scheme is proposed by integrating MIA and GIS to solve and analyze the proposed optimization model. By comparing the optimization results with the current situation, the rationality of the optimization scheme is illustrated. In addition, the superiority of MIA in this optimization conundrum is discussed by comparing the performance of GA, PSO, IA, and MIA.

The results show that the configuration of existing RCFs is irrational in terms of quantity, scale, and location. The number of existing RCFs of Jing’an District is insufficient, and two of them with unreasonable locations require adjustment. There is a significant gap between the service supply of existing RCFs and the care demands of the elderly. There are six new RCFs required to be added to meet the demands of the elderly. The optimization scheme has improved the equity and efficiency of RCF configuration, increased the profits of investors, and reduced the travel costs of the elderly. Compared with conventional algorithms such as the GA, PSO, and IA, the MIA performs better in calculating efficiency and accuracy for solving RCF optimization problems.

The novelty of this paper is that an effective method to optimize the RCF configuration under complex constraints by integrating intelligent algorithms and GIS is proposed. The MIA is an effective algorithm for solving complex optimization problems, while GIS is used to overlay spatial variables to make a composite map. The integration allows the two techniques to mutually benefit from each other and can optimize the quantity, scales, and locations of RCFs simultaneously. We make some contributions in four aspects: Firstly, a multi-objective spatial optimization model is developed for RCF configuration by considering the demands of multiple stakeholders, which enriches the connotation of multi-objective constraints. Secondly, a modified immune algorithm is proposed by improving the standard IA, thus that it can solve optimization problems more efficiently. Thirdly, by integrating the intelligent algorithm and GIS, dynamic optimization, and spatial visualization for RCF configuration is realized. This method also can be used for the configuration optimization of other public service facilities, which broadens the application research of complex spatial decision theory and swarm intelligence optimization theory in the optimization of public service facilities. Finally, the conclusions drawn from this study provide a basis for the planning and configuration decision-making of RCFs in Shanghai and also have reference value for the configuration of RCFs in other cities in China and other developing countries, which develops the theory and method of public service facility planning system. Nevertheless, there are still several limitations in this study. On the one hand, the spatial optimization of the RCF configuration is an intricate multi-objective decision-making problem. Although the objectives of equity, efficiency, cost, and profits have been taken into account in this study, more complex objectives such as policy and resource constraints are possible ones in need of consideration, thus that the spatial optimization model is more realistic. On the other hand, China’s aging process is accelerating and the elderly population is growing rapidly. It is necessary to scientifically predict the elderly populations in the future and their spatial distribution, and propose an optimal configuration plan for RCFs based on the population prediction.

## Figures and Tables

**Table 1:** Descriptive statistics of accessibility to the optimized RCFs.

*β*	Accessibility		
	Maximum	Minimum	Standard Deviation
0.8	2.3879	0.3917	0.6854
1	4.1041	0.4799	0.5018
1.2	7.2491	0.3702	0.8662
1.4	12.4549	0.2694	1.4351
1.6	20.2442	0.1849	2.2596
1.8	30.8292	0.1206	3.3662
2	44.0078	0.0756	4.7404
2.2	59.2022	0.0460	6.3264
2.4	75.5945	0.0275	8.0405

*β* is Distance-decay Parameter.

**Table 2:** The comparison of objective function values for different numbers of RCFs.

The Number of RCFs	4	5	6	7	8	9	10	11	12	13	14
**Objective Function Value**	0.6722	0.6323	0.6012	0.6283	0.6501	0.6766	0.7149	0.7364	0.7626	0.7943	0.8243

**Table 3:** The numbers of beds in newly-added RCFs.

RCF No.	1	2	3	4	5	6
**The number of beds**	285	181	257	247	143	80

**Table 4:** Comparison of optimized results with the current situation.

Objectives	Results		
	Current Situation	Optimized Scheme	Optimization Rate
Equity	56.2886	17.2883	64.23%
Efficiency (%)	—	84.24	84.24%
Travel cost (m/person)	544.9192	389.4611	18.53%
Profits of investor (RMB/month/RCF)	111,785	145,120	29.82%
Gini coefficient	0.5563	0.1225	77.98%
Max travel distance to nearest RCF (m)	2150	1374	36.09%
Average travel distance to nearest RCF (m)	526	373	29.09%
The average number of beds available within one-hour service radius (beds/person)	1.5624	3.3986	117.52%

**Table 5:** Comparison of optimization results obtained by different algorithms.

Objectives	Results			
	GA	PSO	IA	MIA
Equity (number of beds/person)2	19.6840	20.5275	18.2745	17.2883
Efficiency (%)	82.41	83.14	83.67	84.24
Travel cost (m/person)	408.4216	424.4999	411.8927	389.4611
Profits of investor (RMB/ month)	141,970	138,940	145,120	145,120
Comprehensive objective value	0.7323	0.8064	0.6401	0.6012
Number of iterations	650	680	700	480
